# The Small Molecules of Plant Origin with Anti-Glioma Activity

**DOI:** 10.3390/ijms26051942

**Published:** 2025-02-24

**Authors:** Xin Liu, Yin-Xiao Su, Yun-Mei Yang, Rong-Tao Li, Zhi-Jun Zhang

**Affiliations:** Faculty of Life Science and Technology, Kunming University of Science and Technology, Kunming 650500, China; lx18468258082@126.com (X.L.); suyinxiao030104@163.com (Y.-X.S.); yangyang1232025@163.com (Y.-M.Y.)

**Keywords:** natural products, cancer, anti-gliomas, curcumins, resveratrol

## Abstract

Gliomas, originating from glial cells, are prevalent and aggressive brain tumors with high recurrence rates and poor prognosis. Despite advancements in surgical, radiation, and chemotherapeutic treatments, the survival rates remain low. Current standard therapies, such as Temozolomide, have limitations due to cytotoxicity, restricted effectiveness, and severe side effects. So, the development of safer anti-glioma agents is the need of the hour. Bioactive compounds of plant origin, either natural or synthetic, have potential implications due to them actively attacking different targets with a wide range of bioactivities, including anti-glioma activities. In this review, for the first time, there is an overall overview of 51 small molecules of plant origin and seven of their synthetic derivatives, represented as anti-glioma agents in the past decades. The goal of the present review is to provide a summary to comprehend the anti-glioma effects of these compounds in addition to providing a reference for preclinical research into novel anti-glioma agents for future clinical application.

## 1. Introduction

Gliomas, arising from glial cells, represent a significant burden in neuro-oncology due to their high incidence, aggressive nature, and propensity for recurrence. These tumors, which constitute approximately 40% of all central nervous system (CNS) malignancies [1,2], encompass a spectrum ranging from low-grade gliomas to high-grade gliomas, with glioblastoma (GBM) being the most aggressive and prevalent form [3]. Despite advancements in surgical techniques, radiation therapy, and chemotherapeutic interventions, the prognosis for glioma patients remains grim, with low survival rates and frequent relapse [4,5]. The standard-of-care chemotherapeutic agent, Temozolomide (TMZ), while offering some benefit, is limited by its cytotoxicity, restricted efficacy, and the development of drug resistance in glioma cells [6,7].

In the quest for novel therapeutic strategies, natural products (NPs) have emerged as a promising source of anticancer agents. Historically, NPs have played a pivotal role in the discovery and development of numerous medicinal compounds, with over 40% of FDA-approved small molecule anticancer drugs being NP-derived or their derivatives [8]. This rich repository of bioactive compounds continues to inspire research into their potential as anti-glioma agents.

Recent studies have highlighted the efficacy of plant-originated small molecules and their derivatives in combating gliomas. Compounds such as curcumin, resveratrol, magnolol, kaempferol, juglone, tanshinone IIA, nimbolide, and berberine have demonstrated diverse anticancer mechanisms, including apoptosis induction, cell cycle arrest, and signaling pathway modulation. Furthermore, synthetic derivatives of these NPs have shown enhanced bioavailability and anti-tumor efficacy, opening new avenues for glioma therapy [9,10,11].

The present review consolidates recent research on plant-originated small molecules and their derivatives with anti-glioma activities. By exploring the mechanisms underlying their anticancer effects, this review aims to stimulate further research into anti-glioma NP activities and inspire the development of novel drug candidates. By leveraging the diverse bioactivities of NPs, we hope to contribute to the advancement of more effective and less toxic treatment options for glioma patients.

## 2. Anti-Glioma Bioactive Small Molecules of Plant Origin

Anti-glioma bioactive small molecules of plant origin, primarily phenolic acids, quinones, terpenoids, saponins, and alkaloids, derive from diverse plant families like *Zingiberaceae*, *Fabaceae*, and *Celastraceae*. These compounds exhibit versatile anti-glioma activities, underscoring their potential as novel therapeutic agents. The plant resources and targeted cell lines of these anti-glioma active compounds are shown in Table 1.

### 2.1. Phenolic Acids

**Figure 1:**
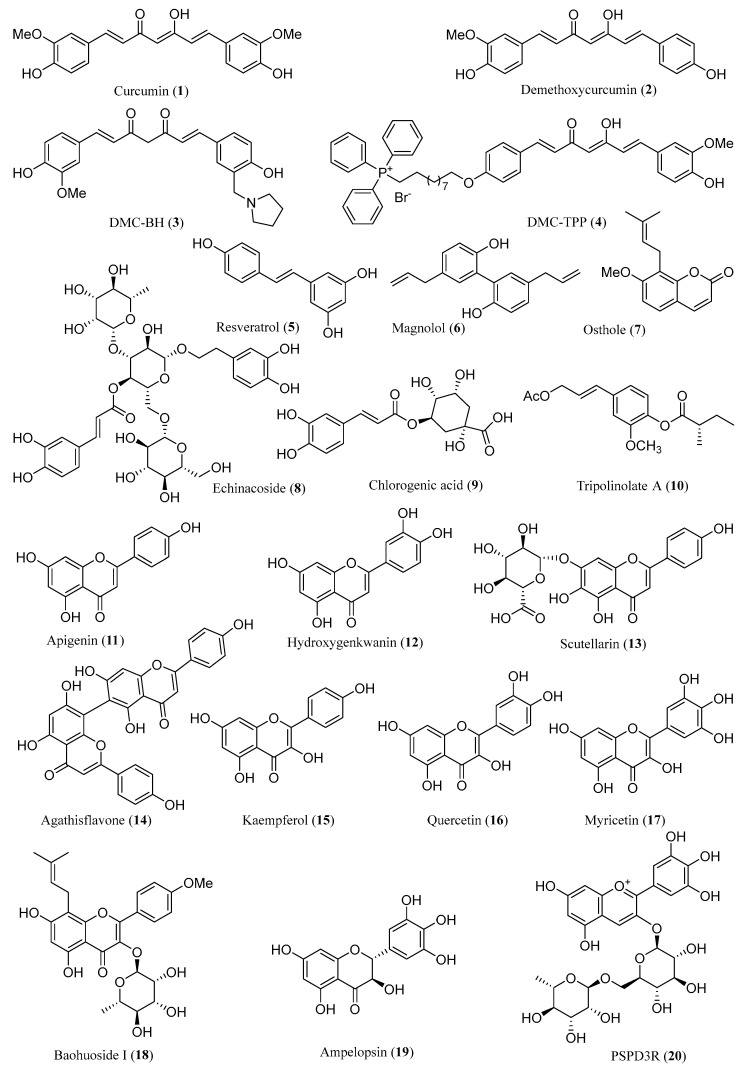
Chemical structures of **1**–**20**.

Phenolic acids, exemplified by curcumin, resveratrol, magnolol, and kaempferol, have emerged as potent anti-glioma agents. These compounds (Figure 1) primarily inhibit glioma cell proliferation, migration, and invasion, while also inducing apoptosis. Their mechanisms involve the cell cycle arrest and modulation of critical signaling pathways, such as the NF-κB, PI3K/Akt/mTOR, ROS-p38-MAPK, JAK/STAT3, and SHH/GLI1 pathways. The research progress on the anti-glioma effect of phenolic acids is as follows.

#### 2.1.1. Curcumins

Curcumin (**1**), a polyphenolic compound abundant in the root of *Curcuma longa*, a member of the *Zingiberaceae* family [12], exhibits a broad spectrum of biological activities and holds promise as a protective agent against various health conditions [13]. Research has demonstrated its ability to inhibit the growth of multiple cancer cell types, including those from the pancreas, breast, colon, lung, leukemia, prostate, and GBM [14,15,16]. In recent years, various synthetic methods have been developed for the preparation of **1**, including traditional approaches and modern methods such as Knoevenagel condensation, the Wittig reaction, and olefin metathesis [17,18]. The use of **1** for treating gliomas has received much attention because of its strong anti-tumor effect and hypotoxicity.

In 2003, it was first discovered to have a significant inhibitory effect on C6 rat glioma 2B-clone cells [19]. A glioma invasion occurs via the complex activities of various matrix metalloproteinase (MMP) molecules. Subsequently, its significantly inhibitory effect on U87MG and U373MG cells for MMPs’ enzymatic activity and protein expression was confirmed [20,21]. G6P translocase (G6PT) is believed to play a crucial role in mediating intracellular signaling events in cancer cells derived from brain tumors. Another study found that G6PT gene expression can be inhibited by **1**, and it can lead to the demise of U-87 glioma cells [22]. Later, the inhibitory effect of it on U251 cells was discovered. The mechanism of action indicates that it induces G2/M phase cell cycle arrest in lower doses, and it not only intensifies this arrest but also triggers S phase arrest in higher concentrations [23]. Another study indicated that it also can inhibit the formation of the side population in C6 glioma cells, which suggests that **1** may be a dietary phytochemical with the potential to target cancer stem cells [24]. Mechanism research shows that it effectively suppresses the proliferation, migration, and invasion of GBM cells by modulating the JAK/STAT3, SHH/GLI1, and Wnt/β-catenin pathways [16,25,26]. Another study has revealed that **1** may combat tumor progression in U87 cells by disrupting the HSP60/TLR4/MYD88/NFκB axis and inducing apoptosis. The inception and progression of GBM have been associated with glioma-initiating cells (GICs), and the results demonstrated that **1** stimulates GIC differentiation and inhibits GIC growth by triggering autophagy [27]. Further research on its underlying mechanism indicated that **1** impeded the transition of the cells from the G1 to the S phase and upregulated the expression of Sox2, Sox4, and Oct4, which were essential to retain the stemness properties of GICs [28]. Additionally, new research demonstrates that **1** reduces the downregulation of Notch1, NEDD4, and pAKT, leading to the inhibition of glioma cell growth, induction of apoptosis, and suppression of invasion and migration [29]. Recent research has demonstrated that **1** effectively represses the growth of U87 and T98 cells, with half-maximal inhibitory concentration (IC_50_) values of 10 μM and 13 μM, respectively [30].

**Figure 2:**
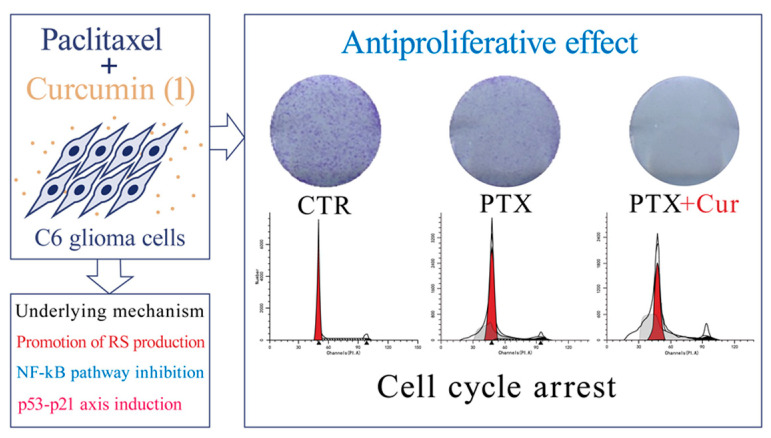
The anti-glioma efficacy of combination therapy of **1** and PTX.

Due to the limited efficacy of monotherapy, current clinical practice in treating cancer includes the use of more molecular-targeted drugs in combination. A study revealed that a combination treatment of **1** and miR-326 causes significant inhibition of the SHH/GLI1 pathway in glioma cells compared with either treatment alone [31]. A subsequent study demonstrated that the combination of curcumin (**1**) and paclitaxel (PTX) effectively suppresses the growth of glioma cells, as confirmed by clonogenic assays and a G0/G1 cell cycle arrest analysis. The combination treatment resulted in a modest yet significant increase in reactive species levels. Furthermore, the drug combination completely inhibited NF-κB nuclear translocation and reduced IκB phosphorylation. Additionally, the results indicated that the combination of the two drugs further enhanced p53 and p21 expression, thereby augmenting the anti-proliferative effects, as shown in Figure 2 [32]. Recent research has shown that low doses of **1** and irradiation exhibit a strong synergistic anti-proliferative effect on both U87 and T98 cell lines in vitro*,* and the combination treatment arrested both cell lines at the G2/M phase to a higher extent than **1** or radiation treatment alone [30].

**Figure 3:**
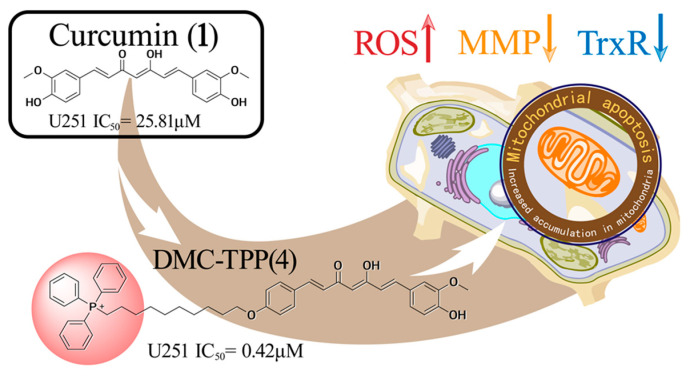
The mechanism of action of **4** triggers mitochondria-dependent apoptosis in U251 cells.

In 2009, research confirmed that demethoxycurcumin (**2**) exhibits anti-proliferative effects on U87 glioma cells by inducing G2/M cell cycle arrest and apoptosis. These effects are associated with the inhibition and downregulation of Bcl-2 [33]. Then, the research showed that it was superior to TMZ in its ability to inhibit proliferation and induce apoptosis of glioma stem cells (GSCs) in vitro. In addition, the combined treatment of TMZ and **2** induced more obvious anti-GSC effects. Mechanistically, they synergistically increase intracellular levels of ROS production, activate the caspase-3-dependent apoptotic pathway, and inactivate the JAK/STAT3 signaling pathway in GBMs [34,35]. However, both **1** and **2** exhibit the disadvantages of poor stability and low bioavailability. To overcome these drawbacks, a series of derivatives of **1** have been synthesized. Among them, **3** exhibited more potent in vitro and in vivo activities compared to **2**. A Western blotting analysis demonstrated that **3** significantly reduced the expression of p-Akt and p-mTOR in orthotopic glioma tissues [9]. Recently, a mitochondria-targeted compound DMC-TPP (**4**) was prepared by conjugating a triphenylphosphine moiety. Biological assays revealed that **4** exhibited heightened cytotoxicity with an IC_50_ value of 0.42 μM against U251 cells and efficient mitochondrial accumulation. Mechanistic investigations suggested (Figure 3) that **4** induces mitochondria-mediated apoptosis, characterized by caspase activation, ROS production, decreased mitochondrial membrane potential, and reduced thioredoxin reductase activity [36].

These findings suggest that **1** holds significant promise as a therapeutic agent for glioma, either as a monotherapy or in combination with conventional treatments, offering a novel approach to combat this malignant tumor.

#### 2.1.2. Resveratrol

Resveratrol (**5**), a phytoalexin naturally present in peanuts, red wine, grapes, multiberries, and other dietary products, has recently attracted the interest of experimental and clinical oncologists [37]. Research has shown that **5** is capable of inducing apoptosis in human glioma U251 cells, potentially through the disruption of cell cycle regulation and the activation of mitochondria-mediated, caspase-3-dependent apoptotic signaling pathways [38]. Another study suggests that **5** inhibited growth and induced cell death in U373 glioma cells [39]. Wang’s team conducted research revealing that **5** effectively restrained glioma cell multiplication by causing cell cycle arrest at the S phase and provoking apoptosis. Delving deeper into the mechanism, they found that **5** significantly decreased the levels of miR-21, miR-30a-5p, and miR-19 [40].

**Figure 4:**
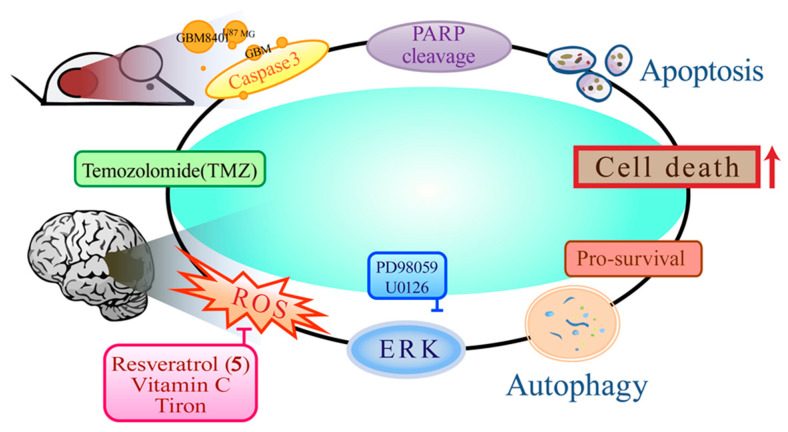
The anti-glioma mechanism of action of combination therapy of **5** and TMZ.

A study demonstrated that TMZ induces both apoptotic cell death and cytoprotective autophagy via a reactive oxygen species (ROS) burst and extracellular signal-regulated kinase (ERK) activation. **5** suppresses this process, leading to decreased autophagy and increased apoptosis. Furthermore, an in vivo mouse xenograft study revealed that the coadministration of **5** and TMZ reduced tumor volumes by inhibiting ROS/ERK-mediated autophagy and subsequently promoting apoptosis (Figure 4) [41]. Additionally, the presence of **5** forces TMZ-treated cells to undergo mitosis, leading to mitotic mutations and aging, reducing the clonal ability of glioma cells, and increasing the chronic effects of TMZ [42]. Recently, the pharmacokinetic and brain distribution studies in mice demonstrated that the PNPs significantly enhanced the bioavailability of paclitaxel-**5** PNPs more than pure paclitaxel and **5**, which led to the conclusion that the paclitaxel-**5** PNPs combination could significantly enhance anti-glioma activity [43]. These results underscore the promising potential of **5** as a therapeutic agent for the management of glioma.

#### 2.1.3. Phenylpropanoids

**Figure 5:**
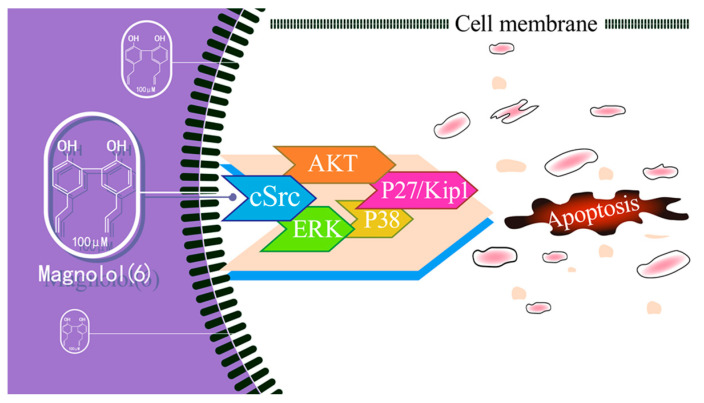
The mechanism of action of **6** triggers apoptosis.

Magnolol (**6**), derived from *Magnolia officinalis*, exhibits potent anticancer properties [44]. Notably, its exceptional ability to traverse the blood–brain barrier (BBB) [45] combined with its neuroprotective effects highlight its safety for non-tumorous brain tissue [46]. A previous study demonstrated that at a concentration of 100 μM, **6** induced apoptosis in U373 cells through the cSrc-mediated upregulation of p27/Kip1 (Figure 5) [47,48]. Additionally, studies have shown that **6**’s anti-GBM effect may be due to inactivating the PKCδ/STAT3 signaling pathways [49], triggering cytotoxic autophagy in glioma cells by suppressing PI3K/AKT/mTOR [50] and enhancing TMZ’s pro-apoptotic effect via NF-κB modulation [51].

Osthole (**7**), a naturally occurring coumarin compound derived from *Cnidium monnieri*, has been extensively studied for its significant anti-tumor properties [52,53]. Ding et al. found that **7** inhibits the growth of C6 glioma cells through the regulation of Mitogen-activated protein kinase (MAPK) and PI3K-Akt signaling pathways [54]. Lin’s team showed that it inhibits the proliferation of U87 glioma cells and induces apoptosis by upregulating miR-16 expression and downregulating MMP-9 [55]. Wiadro and collaborators discovered that **7** primarily induces apoptosis in T98G and MOGGCCM cells, a process linked to the formation of the Bcl-2/Beclin 1 complex [56]. Huangfu’s study proposed that **7** induces necroptosis in glioma cells by ROS generation, with the ROS-mediated RIP1/RIP3/MLKL pathway playing a crucial role in this process [57].

Echinacoside (**8**), a phenylethanoid compound derived from the stalks of *Cistanches salsa*, has a longstanding application in the therapy of malignant tumors. A study on its application in the therapy of malignant tumors revealed that **8** effectively repressed the proliferation, invasive capabilities, and movement of GBM cells while also curbing the growth of xenografts in nude mice models. The underlying mechanism behind its anti-tumor action was attributed to the suppression of the skp2-induced epithelial–mesenchymal transition process [58].

Chlorogenic acid (**9**), commonly derived from traditional Chinese medicinal sources, exhibits diverse pharmacological properties [59]. A study indicated that the SRC/MAPK signaling pathway may be involved in the therapeutic mechanisms of **9** in glioma cells. Moreover, it was discovered that **9** restrains GBM progression both in vivo and in vitro, possibly by suppressing M2 polarization and redirecting macrophage polarization toward an M1-like state, activating STAT1 and inhibiting STAT6 activation [60]. **9** has successfully undergone a phase I clinical trial and is currently in phase II trials for glioma patients, with the initial phase indicating safety, tolerability, and potential anti-tumor efficacy [61]. Recent studies disclosed its capacity to induce apoptosis, cell cycle arrest, and hinder migration and invasion in U373 cells. The IC_50_ values for **9** after 24, 48, and 72 h of exposure were found to be 139.3, 118.6, and 107.3 μM, respectively [62].

Tripolinolate A (**10**), identified initially in *Tripolium pannonicum*, exhibited significant inhibitory effects on the growth of various glioma cell lines, including U251, U87MG, SHG44, and C6, with IC_50_ values ranging from 7.97 to 14.02 μM. It demonstrated substantial anti-glioma activity in nude mice models implanted with U87MG cells. The underlying mechanism revealed that **10** provoked apoptosis in glioma cells by suppressing pro-apoptotic protein expression and arrested the cell cycle at the G2/M phase through the modulation of cell cycle regulators. Moreover, it significantly lowered the expression of several glioma-linked metabolic enzymes and transcription factors [63].

#### 2.1.4. Flavonoid and Glycosides

Flavonoids, a class of naturally occurring compounds prevalent in various herbal remedies, have exhibited significant anti-tumor properties [172], the ability to reverse multi-drug resistance [173], and enhanced permeability across the BBB [174].

Apigenin (**11**), a flavonoid that was extracted from the aerial part of *Portulaca oleracea* L. [64], has garnered substantial interest among researchers due to its diverse biological activities [65]. Hydroxygenkwanin (**12**), with antioxidant properties, has been demonstrated to play a role in preventing blood clot formation [67]. Wang discovered that both compounds **11** and **12** exhibited inhibitory effects on C6 glioma cell growth, with **12** demonstrating more potent anti-glioma action. When administered jointly (**11**+**12**), their anti-glioma efficacy was notably amplified. Functionally, compound **12** restrains the proliferation of C6 glioma cells by activating caspase-3 and caspase-8 via tumor necrosis factor-alpha, thereby inducing apoptosis. Additional mechanisms, such as the mitochondrial apoptotic pathway, DNA damage, and cell cycle arrest, also contribute to this process. In the synergistic effect of **11** + **12**, **12** acts as a facilitator [66].

Scutellarin (**13**) is an active molecule existing in *Erigeron breviscapus* (vant.) Hand-Mazz [68]. Recently, research found that **13** has significant anti-glioma effects. Mechanism studies suggest that **13** can induce apoptosis in glioma cells and suppress proliferation by downregulating BIRC5 expression [69]. Recent research has suggested that agathisflavone (**14**) not only exerts a direct anti-glioma effect but also modulates mesenchymal stem cell responses, thereby influencing GBM proliferation and invasiveness [70]. Kaempferol (**15**) is abundantly present in numerous edible plants, including tea, a majority of vegetables, and fruits. Sharma and colleagues explored the impact of **15** on human GBM cells, discovering that it triggers apoptosis in these cells by escalating ROS production [71]. Siegelin and associates demonstrated that **15** renders U251 and U87 glioma cells more susceptible to TRAIL-induced apoptosis, proposing that this effect is partly due to the flavonoid’s reduction in survivin protein levels [72]. Additional findings propose that **15** induces apoptosis in human glioma cells via caspase-dependent pathways, implicating the modulation of XIAP and survivin by ERK and Akt [73]. Chen’s study reveals that **15** effectively restrains the growth of GBM with minimal toxic or adverse effects [74].

Quercetin (**16**), abundant in various herbs, has demonstrated its efficacy as an anticancer substance, particularly in glioma cases. Notably, Jakubowicz-Gil and associates revealed that Temodal and **16** are synergistic inducers of programmed cell death, better together than applied separately [75]. Another study indicates that **16** and its derivatives possess the ability to traverse the BBB and access the CNS, a critical factor for glioma therapy [76]. Regarding its mechanism of action, **16** suppresses glioma cell growth by modulating both apoptosis and autophagy [77]. Myricetin (**17**) is widely found in many natural plants including bayberry. Numerous prior investigations have uncovered the broad spectrum of anti-tumor impacts of **17** on a wide range of malignancies, including those affecting the esophagus, lungs, leukemia, and prostate cancer cells [78,79]. Li’s study disclosed that the cytotoxic effect of **17** on U251 human glioma cells displayed both dosage and time dependencies. Moreover, it was elucidated that the anti-proliferative action of **17** on these glioma cells is mediated through mitochondrial apoptosis induction, cell cycle arrest at the G2/M phase, the production of ROS, and the suppression of cellular migration [80].

Baohuoside I (**18**), a natural compound extracted from *epimedium*, has demonstrated significant potential in treating a broad spectrum of health conditions [81]. Guo and colleagues illuminated its impact on glioma cell proliferation, motility, and apoptosis, revealing that the compound’s inhibitory action on glioma is mediated by modulating the mTOR signaling pathway, thereby triggering tumor cell apoptosis [82]. Ampelopsin (**19**), a flavonoid component isolated from *Ampelopsis grossedentata* [83], effectively repressed the proliferation of human U251, A172, and SHG44 glioma cells in both a time-sensitive and dosage-dependent manner. Mechanism research shows that **19** caused cell cycle arrest, triggered apoptosis, and stimulated autophagy, thereby impeding glioma progression. These effects were possibly mediated through the production of ROS and JNK phosphorylation [84]. Research has uncovered that PSPD3R (**20**), a compound derived from purple sweet potato, possesses the ability to curb glioma cell multiplication and trigger excessive autophagy and apoptosis through the manipulation of microRNAs. Mechanistic investigations have shown that **20** suppresses the PKB/AKT signaling pathway and influences the dephosphorylation of cyclic adenosine monophosphate-responsive element-binding protein. It also downregulates miR-20b-5p expression, enhances autophagy-related gene 7 activity, and promotes LC3-II conversion, collectively resulting in the overstimulation of autophagy and apoptosis within glioma cells [85].

### 2.2. Quinones

**Figure 6:**
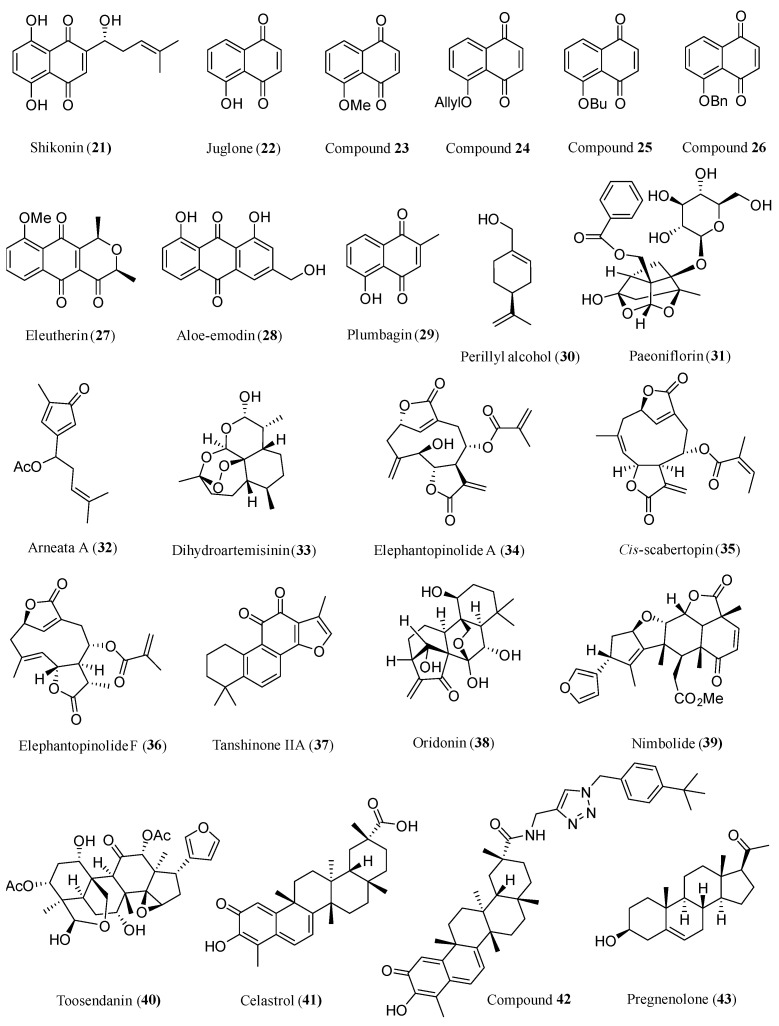
Chemical structures of **21**–**43**.

Naphthoquinones constitute a significant category of naturally occurring compounds, known for their diverse therapeutic activities, including cancer-fighting, antibacterial, and antimalarial effects [175]. The quinone structures form the basis of numerous clinically employed anticancer medications, such as doxorubicin, mitomycin, mitoxantrone, daunorubicin, and saintopin. Numerous naphthoquinones originating from nature exhibit promising potential for advancement as anticancer therapeutics [176]. The structure of anti-glioma active compounds **21**–**29** is shown in Figure 6.

**Figure 7:**
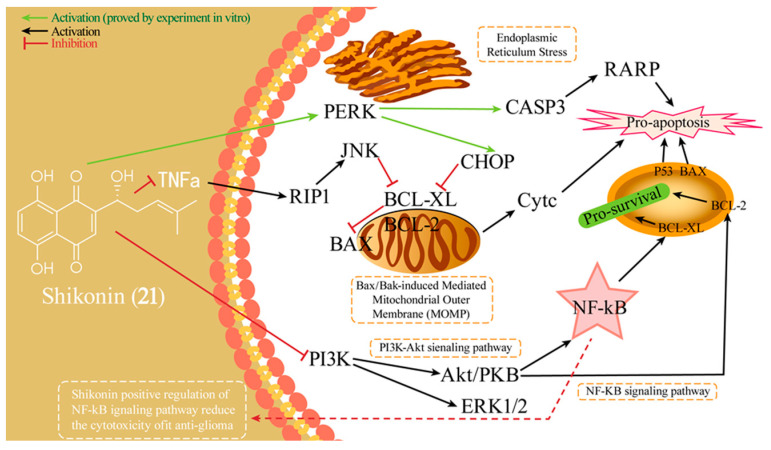
The anti-glioma mechanism of action of **21**.

Shikonin (**21**), isolated from *Lithospermum erythrorhizon* Sieb. et Zucc., is a traditional remedy for various inflammatory and infectious conditions and is also renowned for its diverse anticancer properties [86]. Additionally, it exhibits moderate BBB permeability (BBB = −0.65), high oral bioavailability (OB = 64.79%), and favorable drug-like properties (DL = 0.20) [87]. Research has shown that **21** exhibits a strong inhibitory effect on the proliferation of C6, U87, and U251 cell lines [88,89]. Huang and Ge’s investigation pointed out that **21**-induced demise in glioma cells predominantly occurs by necroptosis induction, with both the RIP-1 pathway and oxidative stress playing contributory roles [88]. Another study has shown that **21** effectively inhibited GSCs and glioma cell viability, arrested the cell cycle at the G0/G1 and S phase, and induced apoptosis through downregulating Bcl-2 and activating the caspase 9/3-dependent pathway [89]. Yang’s research proposed that **21** triggers apoptosis in glioma cells, a process augmented by increased ROS production [90]. Network pharmacology research (Figure 7) showed that **21** effectively targets gliomas by modulating apoptosis pathways. It promotes ER-related and Bax/Bak-induced MOMP apoptosis, enhancing caspase-3 expression in human oligodendroglioma cells in vitro. Simultaneously, it activates pro-survival PI3K-Akt and NF-κB signaling. When combined with anti-GD2 mAb, **21** upregulates PERK and CHOP expression in these cells [87]. Furthermore, Zhang et al. examined the influence of **21** on human GBM cell migration and invasion, discovering that it repressed these processes by dampening MMP-2 and MMP-9 expression and activity [91].

Juglone (**22**), a primary compound found in Juglans mandshurica, demonstrates extensive anticancer effects [176]. A studies have revealed its capacity to suppress the proliferation and decrease the invasive nature of C6 rat glioma cells [92]. Moreover, another study discovered that **22** could inhibit the proliferation of glioma cells with an IC_50_ value of 40 μM and induce apoptosis in glioma stem-like cells by regulating the ROS-p38-MAPK signaling pathway [93]. Nevertheless, **22**’s instability and limited ability to cross the BBB limit its therapeutic potential for brain tumors. Consequently, a range of derivatives of **22** were successfully synthesized. Among them, four of these derivatives (**23**–**26**) displayed potent cytotoxicity against U251 and U87 cells, with IC_50_ values ranging from 3.28 to 11.84 μM and 5.43 to 18.05 μM, respectively. Additional exploration into the underlying molecular mechanisms indicated that these derivatives could trigger apoptosis through ROS generation [10].

Eleutherine (**27**), isolated from the bulbs of *Cipura paludosa*, exhibited significant cytotoxicity against glioma (U-251) cells, with IC_50_ values ranging from 2.6 to 13.8 mg/mL [94]. Recent research has confirmed that **27** reduces C6 cell proliferation in a dose-dependent manner, suppresses migration and invasion, induces apoptosis, and decreases AKT phosphorylation and telomerase expression [95]. Aloe-emodin (**28**) was found in *Aloe vera* foliage, which has demonstrated the capacity to provoke cell death in multiple cancer cell types [96]. Then, its impact on the rat C6 glioma cell line was scrutinized. The results indicate that the anti-glioma effect of **28** occurs through ERK-independent induction of apoptosis and autophagy, along with ERK inhibition-induced differentiation of glioma cells [97]. Plumbagin (**29**), derived from the roots of *Plumbago zeylanica* [98], exhibits a comparatively higher in vitro BBB penetration ability than its structural analogs lapachol and shikonin. Recent findings suggest that **29** restrains glioma development by targeting NQO1/GPX4-mediated ferroptosis, positioning it as a prospective ferroptosis inducer or glioma therapy [99].

### 2.3. Terpenoids

Terpenoids are a highly diverse class of NPs that have historically provided a rich source for the discovery of pharmacologically active small molecules, such as artemisinin and PTX. The structure of anti-glioma active compounds **30**–**42** is shown in Figure 6.

#### 2.3.1. Monoterpenoids

Perillyl alcohol (**30**), derived from lavender, has been employed orally as a cancer therapy for various types of malignancies [100]. Rajesh et al. found that **30** is an effective radiosensitizer inducing apoptosis of T98G cell lines [101]. Other studies demonstrated that **30** induced the apoptosis of human GBM lines and explanted cells from a GBM patient [102] and inhibited the migration and angiogenesis of a murine GBM line [103]. The maximum tolerated dose was established as 8.400 mg/m^2^ per day, administered orally in four doses, based on findings from phase I and phase II clinical trials [104,105]. Another study indicated that **30** (0.3%) by inhalation was well tolerated, had no toxicity given four times daily in patients with malignant glioma, and clearly had anti-tumor activity [106]. The research conducted by Cho et al. demonstrated that **30** shows significant cytotoxic activity against several TMZ-resistant glioma cells (U251, U87, and LN229). Mechanism of action research found that these effects are mediated by the endoplasmic reticulum stress pathway [107].

Paeoniflorin (**31**), a bioactive compound from *Paeonia lactiflora* Pallas plants, is widely acknowledged for its therapeutic potency [108]. A study shows that **31** inhibited U87 cell proliferation and accelerated cell apoptosis. In this study, treatment with **31** was found to upregulate miR-16 expression and decrease MMP-9 protein levels in U87 cells [109]. Furthermore, it suppresses human glioma cells (U87 and U251) by promoting STAT3 degradation through the ubiquitin-proteasome pathway [110]. Another study discovered that **31** restrains glioma cell growth, movement, and infiltration and provokes G2/M phase arrest and apoptosis by modulating Skp2 expression within these cells [111]. Recent research has found that **31** has remarkable cytotoxic properties against U251 and U87 cells with IC_50_ values of 1.34 ± 0.12 and 1.85 ± 0.14 nM, respectively. Further research utilizing a xenograft mouse model based on U87 cells suggests that the anticancer effects of **31** are linked to the translocator protein 18 kDa and neurosteroid synthesis within glioma cells [112]. Arneata A (**32**), extracted from *Arnebia guttata*, exhibited moderate cytotoxic activity against the U118-MG and U373-MG glioma cell lines, with IC_50_ values of 10.4 and 17.5 μM, respectively [113].

#### 2.3.2. Sesquiterpenoids

Dihydroartemisinin (**33**), derived from the medicinal plant *Artemisia annua*, is an FDA-endorsed and WHO-recommended treatment for malaria, known for its safety and efficacy. Xie et al. reported that the concentration of **33** in the brain was approximately two-fold higher than in plasma, indicating its ability to effectively penetrate the BBB [114]. A study shows that **33** enhances the radio sensitivity of U373MG glioma cells by provoking ROS generation and curbing glutathione-S-transferase activity [115]. Additionally, it has been documented to augment the cytotoxic impact of TMZ in rat C6 glioma cells [116]. Another study has shown that **33** could dose-dependently affect GSCs’ sphere morphology, and further investigation disclosed that **33** selectively restrains proliferation and initiates apoptosis in GSCs by inhibiting p-AKT and activating caspase-3 [117]. Another study has revealed that **33** exhibits cytotoxic properties against U251/CP2/U373 glioma cells with IC_50_ values varying from 1.17 ± 0.08 to 23.57 ± 0.80 μg/mL, which suppresses glioma cell proliferation and improves the tumor suppression effectiveness of TMZ by the induction of autophagy [118].

Recent research has shown that three sesquiterpene lactones, elephantopinolide A (**34**), cis-scabertopin (**35**), and elephantopinolide F (**36**), demonstrated the most potent inhibitory action against glioma U87 cells with IC_50_ values of 4.22 ± 0.14, 4.28 ± 0.21, and 1.79 ± 0.24 μM, respectively. These effects might be attributed to their targeting of GSTP1, the activation of the JNK/STAT3 signaling pathway, and the induction of mitochondrial dysfunction and oxidative stress [119].

#### 2.3.3. Diterpenoids

Tanshinone IIA (**37**) is a derivative of phenanthrene-quinone isolated from *Salvia miltiorrhiza* Burge (Danshen), a widely used Chinese herbal medicine [120], and has been reported to improve BBB permeability and effectively penetrate this barrier [121]. A study found that **37** demonstrated significant growth inhibition (IC_50_ = 100 ng/mL) and effectively induced apoptosis and differentiation in human glioma U251 cells [122]. Additionally, **37** inhibits the growth, diminishes stemness properties, and triggers apoptosis in GSCs, potentially through reducing inflammatory cytokine output and blocking the IL6/STAT3 signaling cascade [123]. Further investigation revealed that **37** restrains glioma cell growth, stimulates autophagy and apoptosis, and counters tumorigenesis by disrupting the PI3K/Akt/mTOR pathway [124]. It was observed that **37** displays anti-proliferative and cytotoxic effects on GBM LN-229 cells with an IC_50_ value of 48.2 ± 4.9 μM [125]. At a concentration of 50 μM, **37** suppresses astrocytoma cell migration and proliferation and induces apoptosis by modulating the Notch-1 pathway, influencing the c-Myc, MMP-9, and Bcl-2 proteins [126]. Moreover, the miR-16-5p/TLN1 axis is partially regulated by **37**, contributing to its anti-proliferative, anti-migratory, and anti-invasive effects in glioma cells [127]. Consequently, **37** may represent a promising candidate for the clinical treatment of gliomas.

Oridonin (**38**), initially isolated from *Rabdosia rubescens*, exhibits a broad spectrum of pharmacological actions. Research findings show that **38** possessed a powerful effect on U251 and U87 cells with IC_50_ values of 30.02 and 15.55 μM, respectively. Furthermore, **38** was found to suppress oxidative phosphorylation and glycolysis in glioma cells by modulating PCK2 expression, subsequently triggering an energy crisis and ROS buildup [128].

#### 2.3.4. Triterpenoids

Nimbolide (**39**), a limonoid present in the leaves and flowers of the neem tree (*Azadirachta indica*) [129], possesses a unique structural makeup characterized by a conventional limonoid framework coupled with an α,β-unsaturated ketone moiety and a δ-lactone ring. Research results show that **39** is a potent cytotoxic agent that exerts anti-proliferative and apoptotic effects in T98G, A172, and U87 cells. It reduces the viability of glioblastoma multiforme cells and inhibits tumor growth by suppressing CDK4/6 activity, which results in retinoblastoma hypophosphorylation and cell cycle arrest. Additionally, it targets hyperactivated growth factor pathways in glioblastoma multiforme, such as the PI3K-Akt, MAP kinase, and JAK-STAT pathways [130].

**Figure 8:**
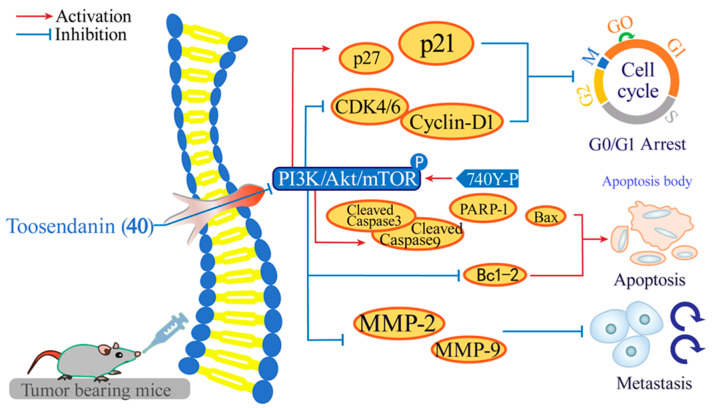
The anti-glioma mechanism of action of **40**.

Toosendanin (**40**), isolated from *Melia azedarach* [131], has demonstrated anti-tumor properties in various cancer cell lines. Zhang and co-workers found that **40** effectively inhibited U87MG, LN18, LN229, and U251 cell proliferation with IC_50_ values of 114.5, 172.6, 217.8, and 265.6 μM, respectively. Mechanism-wise (Figure 8), it inhibits the proliferation, invasion, migration, apoptosis, and cycle distribution of glioma cells by regulating the PI3K/Akt/mTOR signaling pathway [132].

**Figure 9:**
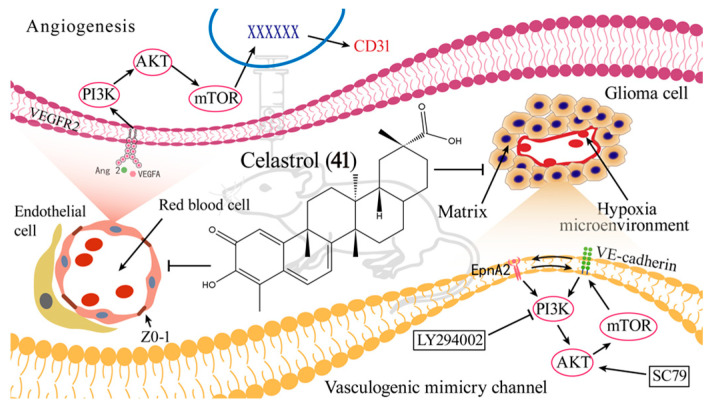
The anti-glioma mechanism of action of **41**.

Celastrol (**41**), a distinctive quinone methide triterpenoid derived from the *Celastraceae* plant family, demonstrates significant potential against various tumors, both in laboratory tests and within living organisms, specifically gliomas [133]. Recent studies have demonstrated that **41** may disrupt vascular structures and promote caveolae-mediated transcellular transport across the BBB. **41** has also been shown to inhibit the proliferation, migration, and invasion of U87 and U251 cells. Moreover, it was discovered (Figure 9) that **41** suppresses vasculogenic mimicry creation and angiogenesis possibly through the modulation of the PI3K/Akt/mTOR signaling cascade [134]. However, its relatively low activity limits its application as an effective agent for glioma treatment. Recently, a range of derivatives of **41** have been designed and synthesized. Subsequently, the inhibitory activity of these compounds on human glioma (A172, LN229, U87, and U251) cell lines was evaluated. Among them, **42** displayed potent anti-proliferative action against the U251 cell lines with an IC_50_ value of 0.94 μM, which is stronger than **41** (IC_50_ = 4.43 μM). Furthermore, **42** notably curbed the colony development and migration of U251 cells. The underlying mechanism disclosed that it triggered necroptosis primarily via activation of the RIP1/RIP3/MLKL pathway. Besides, **42** demonstrated satisfactory permeability across the BBB in mice and significantly repressed U251 cell growth in a zebrafish xenograft model in vivo [11].

### 2.4. Steroids

**Figure 10:**
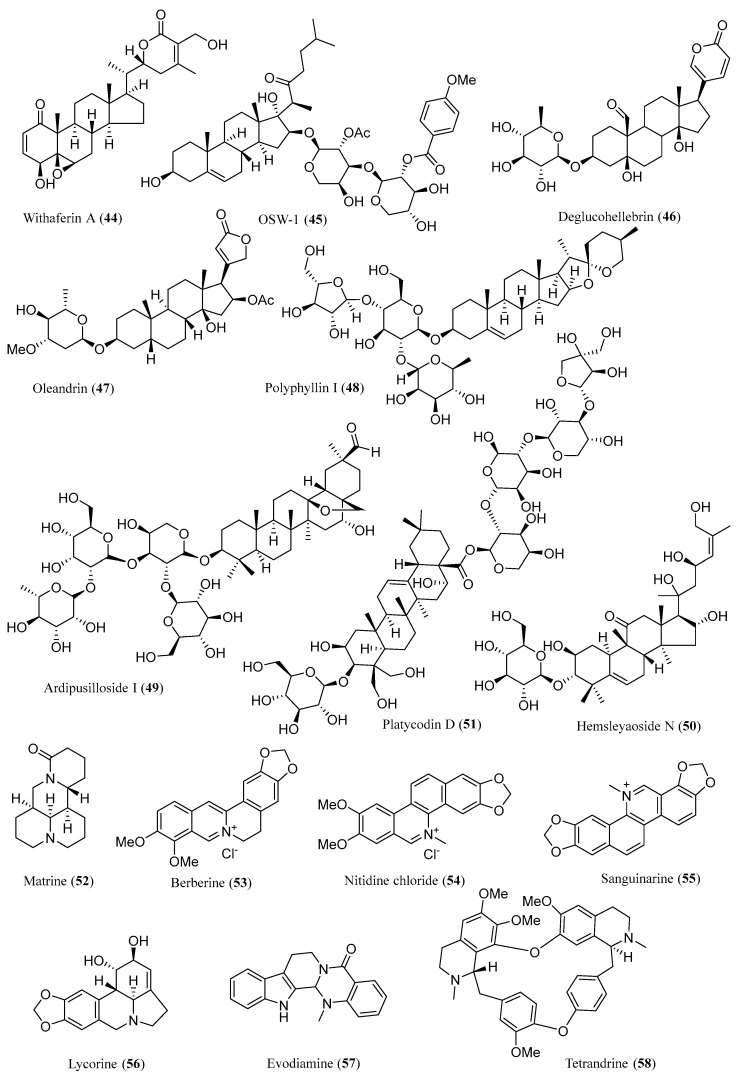
Chemical structures of **44**–**58**.

Pregnenolone (**43**) serves as a fundamental compound present across numerous Chinese herbal remedies. Recent research has disclosed that 100 μM of **43** led to a significant loss in cell viability in various malignant glioma cell (U-87 MG, LN-18, and C6) lines. Furthermore, it has been elucidated that **43** induces glioma cell apoptosis in a caspase-dependent manner, which is mediated by the activation of the extrinsic and intrinsic apoptotic pathways [135]. Due to its highly lipid-soluble chemical structure, **43** can rapidly cross the BBB. Given its potent anti-glioma activity and high BBB permeability, **43** holds promise as a potential therapeutic agent and may facilitate the development of novel anti-glioma therapies. Withaferin A (**44**) exhibits a wide spectrum of pharmacological effects, prominently including anti-tumor activities. Recent research has unveiled its potential to induce apoptosis in C6 glioma cells. Further investigation revealed that its anti-glioma action stems from suppressing C6 cell proliferation (a cytotoxic/anti-proliferative effect) and inflammatory reactions (the inhibition of NF-p65 translocation), ultimately leading to programmed cell death (the activation of a caspase cascade) [136]. Moreover, **44** has been reported to cross the BBB [137], and hence it would act as an effective anti-glioma agent. The structure of **43** is shown in Figure 6 and the structure of **44** is shown in Figure 10.

### 2.5. Saponins

Saponins are plant glycosides with favorable anti-tumorigenic properties. The structure of anti-glioma active compounds **45**–**51** is shown in Figure 10.

#### 2.5.1. Steroid Saponins

Research on the biological properties OSW-1 (**45**) revealed a remarkable cytotoxic potency against malignant cancer cells, outperforming several standard chemotherapy agents like doxorubicin, camptothecin, and paclitaxel [138]. Zhan and Huang delved into the anti-tumor effects of **45** on glioma cells. Their investigation disclosed that the IC_50_ concentrations for T98G cells at 24, 48, and 72 h were 43.35, 13.02, and 0.07 nM, respectively, while for LN18 cells, they were 15.73, 0.45, and 0.04 nM, correspondingly. Further research showed that **45** suppressed cell viability and reproduction, triggered apoptosis, and hindered cell cycle progression at the G2/M stage. Importantly, the inhibition of the PI3K/AKT signaling pathway by **45** was identified as a pivotal mechanism contributing to its anticancer actions [139]. A study found that deglucohellebrin (**46**) was effective in suppressing the viability of GBM cell lines. Furthermore, **46** has a low molecular weight of 562 Daltons, which may facilitate its passage across the BBB. These findings also indicated that **46** caused G2/M phase cell cycle arrest, activated caspase-8, and led to considerable mitochondrial membrane depolarization, which collectively pointed toward the activation of the mitochondria-mediated intrinsic apoptotic pathway [140].

A study confirms that oleandrin (**47**), a polyphenolic cardiac glycoside derived from the leaves of *Nerium oleander* [141], effectively suppressed glioma progression and hindered cellular multiplication. The mechanism behind this effect was partially uncovered as the potency of **47** decreased in brain-derived neurotrophic factor knockout mice and in glioma cells with silenced TrkB expression, suggesting a crucial involvement of BDNF in the therapeutic effectiveness of **47**. Another significant finding was that **47** also improved survival rates and augmented the efficacy of TMZ in mice with implanted gliomas [142]. A recent investigation explored the suppressive impact of polyphyllin I (**48**) on the progression of U251 human glioma cells. It was revealed that **48** effectively repressed the growth of U251 cells with an IC_50_ value of 5.74 μM. Additional studies disclosed that the potent inhibition occurs by cell cycle arrest at the G2/M phase and induction of apoptosis and mitochondrial disturbances, facilitated by the activation of the JNK signaling cascade. Furthermore, the study revealed that **48** also exhibited inhibitory activity against the U87 glioma cell line, indicating a broader applicability across different glioma cell types [143].

#### 2.5.2. Triterpenoid Saponins

Research has shown that ardipusilloside I (**49**), a triterpene-saponin isolated from the traditional Chinese medicine *Ardisia pusilla* A. DC., triggers apoptosis in human GBM cells by the FasL/Fas pathway or induction of autophagy, and it also restrains NCI-H460 cell proliferation by modulating Bcl-2, Bax, and VEGFR expression [144,145]. However, the low oral bioavailability and hemolytic effects of ADS-I remain significant obstacles to its clinical application. Therefore, in a separate study, it was demonstrated that encapsulating **49** within a polymer wafer maintained its inhibitory effects on rat C6 glioma cells. Furthermore, the use of **49**-loaded wafer implants showed significantly greater suppression of C6 glioma growth compared to the same dose of **49** in solution form. This highlights the potential of **49**-containing wafers for effective glioma management [146]. In 2022, twenty-three cucurbitane-type tetracyclic triterpenoids were extracted from the tubers of *Hemsleya penxianensis*. Among these compounds, *hemsleyaoside* N (**50**) demonstrated significant anti-glioma effectiveness in both recurrent GBM cell lines derived from patients and orthotopic nude mouse models. Delving into the underlying mechanism, it was discovered that **50** concurrently activated the unfolded protein response and MAPK pathways, subsequently leading to endoplasmic reticulum stress-induced apoptosis [147]. Recently, platycodin D (**51**), an effective triterpene saponin extracted from the root of *Platycodon grandiflorum*, has been rigorously examined for its impact on GBM [148]. The study reveals that **51** suppresses autophagy and facilitates cell death through increasing the low-density lipoprotein receptor expression levels in U87 and U373 glioma cell lines [149]. Furthermore, another study indicates that **51** restrains the growth, movement, and infiltration of glioma cells, and it influences glioma suppression by modulating the Skp2-p21-p27 signaling pathway [150].

### 2.6. Alkaloids

Matrine (**52**), a compound found in the roots of *Sophora* species [151], exhibits inhibitory effects on the growth of C6 cell lines at various concentrations, demonstrating a clear dose–response relationship. Further investigation revealed that upon exposure to **52**, the C6 cells experienced cell cycle arrest at the G1 phase [152]. Berberine (**53**) exhibits potent anticancer effects against various malignancies, and Eom’s research revealed that it decreases T98G cell viability by triggering apoptosis by endoplasmic reticulum stress and ROS-linked mitochondrial pathways [153]. Specifically, it has been demonstrated to curb the proliferation of T98G, LN18, C6, SHG44, and LN229 cell lines. Mechanistically, it might induce glioma cell apoptosis through ERK1/2-mediated mitochondrial dysfunction and control glioma cell growth by modulating wild-type p53 activation or mutant p53 inhibition [154]. Furthermore, **53** exerts its suppressive action on U87 and U251 cells in a time- and concentration-dependent manner [155]. Recently, it was shown that **53** potentially diminishes glioma cell migration and invasion capabilities by targeting the TGF-b1/COL11A1 signaling axis [156]. In addition, BBR can cross the BBB and have a neuroprotective effect on the brain [157,158]. Thus, the natural compound BBR can potentially be used in the future to treat gliomas.

Nitidine chloride (**54**), extracted from *Zanthoxylum nitidum* (Roxb) [159], is well documented for its inhibitory effects on the proliferation of hepatocellular carcinoma and renal cancer cells [160]. Other studies have demonstrated that **54** effectively inhibits the proliferation of U87 and C6 cell lines, with calculated IC_50_ values of 10 and 8 μM, respectively [161]. Recently, it has been demonstrated that this compound induces apoptosis through the activation of caspases 3 and 7. Furthermore, it has been shown to target the PIK3/AKT/mTOR pathway, thereby inhibiting aggressive GBM cells [162]. Pallichankandy and colleagues delved into the molecular underpinnings of autophagy triggered by sanguinarine (**55**) in human malignant glioma cells. Their study disclosed that **55** is efficacious in retarding the proliferation of human malignant glioma cells through autophagic cell death, a process closely tied to the ROS-mediated augmentation of ERK1/2 signaling [163]. A study showed that lycorine (**56**) demonstrates substantial cytotoxic effects on U251 cells, with an IC_50_ value of 10 μM. This study also reveals that **56** effectively suppresses the progression of GBM by dampening the expression levels of the epidermal growth factor receptor and disrupting its downstream signaling cascade through a direct interaction with the receptor [164]. Recent findings suggest a dosage-sensitive inhibition of C6 glioma cell proliferation by **56**. Investigating the underlying mechanisms, it has been determined that the anti-tumor effects of **56** are mediated by the induction of ROS and the disruption of the NF-κB signaling pathway [165]. Of particular importance, **56** is a compound that can effectively penetrate the BBB and does not induce significant CYP3A4 inhibitory activity [166]. This characteristic allows **56** to readily access GBM primary tumors in the cranial cavity via oral administration or intravenous injection, without inducing systemic hepatotoxicity.

Evodiamine (**57**), derived from *Evodia rutaecarpa*, is a staple in traditional Chinese medicine and exhibits diverse biological activities [167]. Earlier studies revealed that **57** caused cell death in human U87-MG GBM cells with an IC_50_ value of 5.21 μM by triggering calcium/JNK-regulated autophagy and calcium/mitochondria-induced apoptosis [168]. Another study suggests that it dose-dependently suppressed GBM cell growth. This study also found that the concurrent use of **57** and tumor necrosis factor-α-related apoptosis-inducing ligand (TRAIL) notably augmented apoptosis in U87 GBM cells by upregulating the expression of DR4 and DR5 receptors [169]. In addition, its impact on U87-MG and C6 cell lines was further investigated, which led to the discovery that **57** could diminish the survival of both GBM cell types through apoptosis induction and cell cycle arrest at the G2/M phase [170]. A study found that tetrandrine (**58**) exerts a dose-responsive suppression on human glioma cell lines, specifically U87, U251, and SWO-38. This inhibition of tumor progression is achieved through multiple mechanisms, including triggering apoptosis via the mitochondrial pathway, halting the cell cycle at the G0/G1 phase, thereby hindering the proliferation of glioma cells, and also disrupting angiogenesis. More importantly, **58** could decrease the expression of phosphorylated STAT3 and its downstream proteins [171]. The structure of anti-glioma active compounds **52**–**58** is shown in Figure 10.

## 3. Conclusions

Gliomas, the most prevalent primary brain malignancies, are notoriously aggressive and characterized by high recurrence rates and poor prognosis [1,2]. Traditional treatment options, including surgery, radiation therapy, and chemotherapy (such as TMZ), have shown limited success due to issues like cytotoxicity, restricted effectiveness, and severe side effects [6,7]. In recent years, however, significant progress has been made in glioma treatment strategies, particularly with the advent of innovative therapeutic agents and delivery systems. A notable advancement is the utilization of nanotechnology to develop nanocarriers that can effectively transport drugs across the BBB, enhancing the efficacy of glioma treatments. For instance, nanocarriers can encapsulate curcumin, a natural polyphenolic compound with strong anticancer properties, facilitating its delivery directly to the tumor site. This targeted approach not only boosts therapeutic efficacy but also minimizes systemic toxicity. Nevertheless, the development of these nanomedicines presents certain challenges. These include complex manufacturing processes, potential immunogenicity, and the necessity for a thorough evaluation of their biodistribution and toxicity profiles [177,178,179].

NPs encompass all metabolites derived from plants, animals, and microorganisms. Over the past century, structurally diverse NPs have significantly advanced drug discovery, contributing to the development of anti-tumor agents, antibiotics, immunosuppressants, cardiovascular and cerebrovascular medications, and other clinically used drugs, many of which are closely associated with NPs. Compared to synthetic compounds, NPs exhibit greater structural diversity and molecular chirality, as well as a broader range of lipophilicity and molecular weights. Additionally, NPs play an active role in modulating various biological processes. These unique characteristics and advantages place NPs at the forefront of pharmaceutical research. Consequently, NPs and their derivatives have emerged as promising candidates for anti-glioma therapy.

This review consolidates recent research on plant-originated small molecules and their derivatives with demonstrated anti-glioma activities. Compounds such as **1**, **2**, **6**, **15**, **22**, **37**, **39**, and **53** have exhibited a variety of anticancer mechanisms, including apoptosis induction, cell cycle arrest, and signaling pathway modulation [15,16,26]. Furthermore, synthetic derivatives of **1**, **22**, and **41** have shown enhanced bioavailability and anti-tumor efficacy, providing avenues for the development of more effective glioma treatments [9,10,11].

Despite the promising anti-glioma effects of the natural compounds discussed in this review, it is crucial to acknowledge the potential side effects and toxicity issues associated with their use. Natural compounds, while generally considered less toxic compared to synthetic drugs, can still exhibit adverse effects, particularly when administered at high doses or over prolonged periods. For instance, curcumin has been reported to cause gastrointestinal discomfort, such as diarrhea, nausea, and abdominal pain, in some individuals [180]. Additionally, although it has low toxicity, long-term use may lead to liver injury in certain cases [181]. Similarly, resveratrol has been associated with mild adverse effects like headache, nausea, and insomnia, especially at higher doses [182]. Other compounds, like magnolol, osthole, and shikonin, have limited clinical data on their toxicity profiles. However, in vitro and animal studies have suggested potential toxicity at high concentrations. For example, magnolol has been shown to be cytotoxic to non-tumorous cells at high doses [44], and osthole has been associated with embryotoxicity in animal models [52]. Shikonin, while effective against gliomas, has also demonstrated toxicity toward normal cells in some studies [88].

Based on the discussion above, while plant-originated small molecules and their derivatives exhibit promising anti-glioma activities through various mechanisms, it is essential to carefully consider their potential side effects and toxicity issues. Despite generally being less toxic than synthetic drugs, natural compounds can still cause adverse effects, particularly at high doses or with prolonged use. Therefore, thorough preclinical and clinical evaluations are crucial to fully understand their toxicity profiles and ensure safety in clinical applications. Future research should focus on optimizing the bioavailability, potency, and selectivity of these compounds while also exploring strategies to mitigate their potential toxicity. With continued efforts, it is hoped that safe and effective anti-glioma drugs derived from natural products will be developed, ultimately improving the prognosis and quality of life for glioma patients.

## Figures and Tables

**Table 1:** The plant resources and targeted cell lines of anti-glioma active compounds **1**–**58**.

Compound Name	Plant Species	IC_50_/Targeted Cell Lines	Mechanism	References
Curcumin (**1**)	*Curcuma longa*	10 μM (U87), 13 μM (T98)	Inhibits glioma cells by modulating key pathways. Induces apoptosis, stimulates GIC differentiation. Impedes cell cycle, upregulates stemness genes. Reduces growth factors, effective against glioma cell lines	[12,13,14,15,16,17,18,19,20,21,22,23,24,25,26,27,28,29,30,31,32]
Demethoxycurcumin (**2**)	*Curcuma longa*	U87	Synergizes with TMZ to promote apoptosis via ROS and caspase-3, inactivate JAK/STAT3	[33,34,35]
DMC-BH (**3**)	Derivative of **1**	SHG-44, C6, U251, U87, A172	Reduced p-Akt and p-mTOR expression in orthotopic glioma tissue	[9]
DMC-TPP (**4**)	Derivative of **1**	0.42 μM (U251)	Mitochondria-mediated apoptosis, caspase activation, ROS production, decreased mitochondrial membrane potential	[36]
Resveratrol (**5**)	Peanuts, grapes, etc.	U251, U373	Apoptosis induction, cell cycle arrest (S phase), modulation of miR-21, miR-30a-5p, miR-19	[37,38,39,40,41,42,43]
Magnolol (**6**)	*Magnolia officinalis*	100 μM (U373)	Apoptosis induction through cSrc-mediated upregulation of p27/Kip1, inactivation of PKCδ/STAT3 signaling pathways, cytotoxic autophagy, NF-κB modulation	[44,45,46,47,48,49,50,51]
Osthole (**7**)	*Cnidium monnieri*	C6, U87, T98G	Inhibits glioma growth, induces apoptosis via pathway regulation and miR-16/MMP-9 modulation, and triggers necroptosis through ROS-mediated pathway	[52,53,54,55,56,57]
Echinacoside (**8**)	*Cistanches salsa*	U87, U251	Inhibition of glioma cell proliferation, invasion, and migration, suppression of skp2-induced epithelial–mesenchymal transition process	[58]
Chlorogenic acid (**9**)	Coffee beans	107.3 μM (U373)	Inhibits GBM, induces cell death, and reduces migration in U373 cells. Shows therapeutic potential in clinical trials	[59,60,61,62]
Tripolinolate A (**10**)	*Tripolium pannonicum*	7.97-14.02 μM (U251, U87MG, SHG44, C6)	Apoptosis induction, cell cycle arrest (G2/M phase), modulation of glioma-linked metabolic enzymes and transcription factor	[63]
Apigenin (**11**)	*Portulaca oleracea*	C6	Inhibition of glioma cell growth (in combination with **12**)	[64,65,66]
Hydroxygenkwanin (**12**)	*Daphne genkwa*	C6	Induces apoptosis in C6 glioma cells through caspase activation mediated by tumor necrosis factor-alpha along with other mechanisms and exhibits enhanced anti-glioma efficacy when combined with **11**	[66,67]
Scutellarin (**13**)	*Erigeron breviscapus*	U251	Apoptosis induction, proliferation suppression through downregulation of BIRC5 expression	[68,69]
Agathisflavone (**14**)	*Rhus succedanea*	GL-15, U373	Direct anti-glioma effect, modulation of mesenchymal stem cell responses	[70]
Kaempferol (**15**)	Various edible plants	U251, U87	Induces apoptosis in glioma cells by increasing ROS, enhancing TRAIL sensitivity, and modulating XIAP/survivin via ERK/Akt pathways, with minimal toxicity	[71,72,73,74]
Quercetin (**16**)	Various herbs	U87, U251	Modulation of apoptosis and autophagy, synergistic induction of programmed cell death with Temodal	[75,76,77]
Myricetin (**17**)	*Hypericum perforatum*	U251	Exhibits anti-proliferative effects through mitochondrial apoptosis, G2/M arrest, ROS production, and migration inhibition in glioma cells	[78,79,80]
Baohuoside I (**18**)	*Epimedium brevicornu*	U251	Modulation of the mTOR signaling pathway, apoptosis induction	[81,82]
Ampelopsin (**19**)	*Ampelopsis grossedentata*	U251, A172, SHG44	Inhibits glioma proliferation through cell cycle arrest, apoptosis, and autophagy, mediated by ROS and JNK phosphorylation	[83,84]
PSPD3R (**20**)	*Ipomoea batatas*	U251, A172	Curbs glioma proliferation by manipulating microRNAs, suppressing PKB/AKT, influencing CREB, downregulating miR-20b-5p, enhancing ATG7, promoting LC3-II conversion	[85]
Shikonin (**21**)	*Lithospermum erythrorhizon*	C6, U87, U251	Inhibits glioma via necroptosis, apoptosis, enhances caspase-3, activates signaling pathways. Upregulates PERK, CHOP. Reduces migration, invasion	[86,87,88,89,90,91]
Juglone (**22**)	*Juglans mandshurica*	40 μM (U87)	Inhibition of proliferation, apoptosis induction through ROS-p38-MAPK signaling pathway	[92,93]
Compound **23**–**26**	Derivatives of **22**	3.28-18.05 μM (U251, U87)	Apoptosis induction through ROS generation	[10]
Eleutherine (**27**)	*Cipura paludosa*	2.6-13.8 mg/mL (U-251)	Inhibition of proliferation, migration, and invasion, apoptosis induction, decreased AKT phosphorylation, telomerase expression	[94,95]
Aloe-emodin (**28**)	*Aloe vera* foliage	C6	Apoptosis and autophagy induction through ERK-independent pathways, ERK inhibition-induced differentiation	[96,97]
Plumbagin (**29**)	*Plumago zeylanica*	U87, U251, C6, T98G, LN18	Ferroptosis induction through NQO1/GPX4 pathway	[98,99]
Perillyl alcohol (**30**)	*Cymbopogon citratus*	T98G, U251, U87, LN229	Exhibits anti-tumor activity; induces apoptosis, inhibits migration/angiogenesis; cytotoxic to TMZ-resistant cells via ER stress pathway	[100,101,102,103,104,105,106,107]
Paeoniflorin (**31**)	*Paeonia lactiflora*	1.34 nM (U251), 1.85 nM (U87)	Inhibits proliferation, promotes apoptosis, reduces mobility/invasion. Upregulates miR-16, decreases MMP-9, promotes STAT3 degradation, induces G2/M arrest. Linked to TP18kDa, neurosteroid synthesis	[108,109,110,111,112]
Arneata A (**32**)	*Arnebia guttata*	10.4 μM (U118-MG), 17.5 μM (U373-MG)	Moderate cytotoxic activity	[113]
Dihydroartemisinin (**33**)	*Artemisia annua*	U373MG, C6, U251, CP2, U373	Enhances radio-sensitivity. Augments TMZ effect. Inhibits GSC, induces apoptosis. Cytotoxic, enhances TMZ via autophagy	[114,115,116,117,118]
Elephantopinolide A (**34**)	*Elephantopus scabe*r	4.22 μM (U87)	Target GSTP1, activate JNK/STAT3, induce mitochondrial dysfunction, oxidative stress	[119]
Cis-scabertopin (**35**)	*Elephantopus scabe*r	4.28 μM (U87)	Target GSTP1, activate JNK/STAT3, induce mitochondrial dysfunction, oxidative stress	[119]
Elephantopinolide F (**36**)	*Elephantopus scabe*r	1.79 μM (U87)	Target GSTP1, activate JNK/STAT3, induce mitochondrial dysfunction, oxidative stress	[119]
Tanshinone IIA (**37**)	*Salvia miltiorrhiza*	100 ng/mL (U251), 48.2 μM (LN-229)	Inhibits glioma growth, induces apoptosis. Reduces GSC stemness, disrupts PI3K/Akt/mTOR. Anti-proliferative on GBM, suppresses migration. Influences Notch-1, regulates miR-16-5p/TLN1	[120,121,122,123,124,125,126,127]
Oridonin (**38**)	*Rabdosia rubescens*	30.02 μM (U251), 15.55 μM (U87)	Suppresses oxidative phosphorylation, glycolysis via PCK2 modulation. Triggers energy crisis, ROS buildup	[128]
Nimbolide (**39**)	*Azadirachta indica*	T98G, A172, U87	Anti-proliferative, apoptotic in T98G, A172, U87. Reduces GBM viability, inhibits growth via CDK4/6 suppression. Targets PI3K-Akt, MAP kinase, JAK-STAT pathways	[129,130]
Toosendanin (**40**)	*Melia azedarach*	114.5 μM (U87MG), 172.6 μM (LN18), 217.8 μM (LN229), 265.6 μM (U251)	Inhibits proliferation, invasion, migration, apoptosis, cycle distribution. Acts by regulating the PI3K/Akt/mTOR signaling pathway	[131,132]
Celastrol (**41**)	*Celastraceae*	4.43 μM (U251)	Inhibits proliferation, migration, invasion. Suppresses vasculogenic mimicry and angiogenesis via PI3K/Akt/mTOR	[133,134]
Compound **42**	Derivative of **41**	0.94 μM (U251)	Potent anti-proliferative, curbs colony and migration. Triggers necroptosis via RIP1/RIP3/MLKL	[11]
Pregnenolone (**43**)	*Auricularia auricula*	100 μM (U-87MG, LN-18, C6)	Induces apoptosis via caspase-dependent pathways	[135]
Withaferin A (**44**)	*Withania somnifera*	C6	Suppresses proliferation and inflammation, leads to cell death via caspase	[136,137]
OSW-1 (**45**)	*Ornithogalum saundersiae*	0.07 nM (T98G); 0.04 nM (LN18)	Suppresses viability, triggers apoptosis. Hinders G2/M cell cycle. Inhibits PI3K/AKT pathway	[138,139]
Deglucohellebrin (**46**)	*Helleborus odorus*	U251MG, T98G	Induces G2/M arrest, caspase-8 activation, and mitochondrial depolarization, triggering intrinsic apoptosis	[140]
Oleandrin (**47**)	*Nerium oleander*	GL261, U87MG, A172, U251, GL15	Suppresses glioma, hinders multiplication. BDNF/TrkB-dependent. Enhances TMZ efficacy	[141,142]
Polyphyllin I (**48**)	*Paris polyphylla*	5.74 μM (U251)	Inhibits via G2/M arrest, apoptosis, JNK activation	[143]
Ardipusilloside I (**49**)	*Ardisia pusilla*	NCI-H460, C6	Triggers apoptosis in GBM cells via FasL/Fas or autophagy. Restrains NCI-H460 cell proliferation by modulating Bcl-2, Bax, VEGFR	[144,145,146]
Hemsleyaoside N (**50**)	*Hemsleya penxianensis*	U118-MG, U251-MG	Activates UPR and MAPK, inducing ER stress apoptosis	[147]
Platycodin D (**51**)	*Platycodon grandiflorum*	U87, U373	Suppresses autophagy, enhances cell death via LDLR. Inhibits glioma growth and movement Modulates Skp2-p21-p27 pathway	[148,149,150]
Matrine (**52**)	*Sophora flavescens*	C6	Inhibition of proliferation, cell cycle arrest (G1 phase)	[151,152]
Berberine (**53**)	*Berberis vulgaris*	T98G, LN18, C6, SHG44, LN229, U87, U251	Triggers apoptosis via ER stress and ROS. Inhibits proliferation, modulates p53. Suppresses cells in time/dose-dependent manner. Diminishes migration/invasion by targeting TGF-b1/COL11A1	[153,154,155,156,157,158]
Nitidine chloride (**54**)	*Zanthoxylum nitidum*	10 μM (U87), 8 μM (C6)	Induces apoptosis via activation of caspases 3 and 7. Targets PIK3/AKT/mTOR pathway to inhibit aggressive GBM cells	[159,160,161,162]
Sanguinarine (**55**)	*Sanguinaria canadensis*	U87MG, U118MG	Retardation of proliferation through autophagic cell death, ROS-mediated ERK1/2 signaling augmentation	[163]
Lycorine (**56**)	*Amaryllidaceae*	10 μM (U251)	Suppresses GBM via EGFR downregulation. Inhibits C6 proliferation in a dose-dependent manner. Acts via ROS induction and NF-κB disruption	[164,165,166]
Evodiamine (**57**)	*Evodia rutaecarpa*	5.21 μM (U87-MG)	Induces cell death in GBM cells via autophagy and apoptosis. Suppresses GBM growth in a dose-dependent manner. Enhances TRAIL-induced apoptosis. Diminishes GBM cell survival through apoptosis and G2/M arrest	[167,168,169,170]
Tetrandrine (**58**)	*Stephania tetrandra*	U87, U251, SWO-38	Suppresses glioma cells, induces apoptosis, arrests cell cycle, disrupts angiogenesis. Decreases p-STAT3 expression	[171]

## Abbreviations

CNS	Central nervous system
GBM	Glioblastoma
TMZ	Temozolomide
NPs	Natural products
MMP	Matrix metalloproteinase
G6PT	G6P translocase
GICs	Glioma-initiating cells
IC_50_	Half-maximal inhibitory concentration
PTX	Paclitaxel
GSCs	Glioma stem cells
ROS	Reactive oxygen species
ERK	Extracellular signal-regulated kinase
BBB	Blood–brain barrier
MAPK	Mitogen-activated protein kinase
